# sEMG dataset of routine activities

**DOI:** 10.1016/j.dib.2020.106543

**Published:** 2020-11-19

**Authors:** Asad Mansoor Khan, Sajid Gul Khawaja, Muhammad Usman Akram, Ali Saeed Khan

**Affiliations:** Department of Computer and Software Engineering, CEME, National University of Sciences and Technology, Islamabad, Pakistan

**Keywords:** sEMG, IMU, Physical actions, Routine activities, Accelerometer, Gyroscope

## Abstract

In this paper, we present the data set of surface electromyography (sEMG) and an Inertial Measurement Unit (IMU) against human muscle activity during routine activities. The Myo Thalamic Armband is used to acquire the signals from muscles below the elbow. The dataset comprises of raw sEMG, accelerometer, gyro and derived orientation signals for four different activities. The four activities, which are selected for this dataset acquisition, are resting, typing, push up exercise and lifting a heavy object. Therefore, there are five associated files against each activity. The IMU data can be fused with the sEMG data for better classification of activities especially to separate aggressive and normal activities. The data is valuable for researchers working on assistive computer aided support systems for subjects with disabilities due to physical or mental disorder.

## Specifications Table

**Table:** 

Subject	Surface Electromyography and Clinical Neurophysiologist
Specific subject area	Physical Actions, sEMG, IMU, Epilepsy
Type of data	Signals Excel Files
How data were acquired	Signals were acquired using Myo Thalamic Armband
Data format	Comma Separated Values (CSV) files containing 1 Raw sEMG data 2 Raw Gyro data 3 Raw Accelerometer data 4 Orientation (Euler/Non-Euler) data derived from Gyro data 5 Folder Nomenclature: It has been divided into two parts; subject_xx where xx is the ID of the subject and file_yy where yy is the action performed by a subject xx.
Parameters for data collection	sEMG signals of routine activities acquired from upper limbs
Description of data collection	Dataset contains signals of routine activities that have been collected from the arm just below the elbow. The dataset is mainly focused on differentiation of daily activities from one another.
Data source location	Biomedical Image/Signal Analysis (BIOMISA) Research Lab National University of Sciences and Technology Rawalpindi Pakistan 33.623482° N, 72.958688° E
Data accessibility	Repository name: Mendeley Data Data identification number: DOI: 10.17632/bcv9vsxkyc.2 Direct URL to data: https://data.mendeley.com/datasets/bcv9vsxkyc/2

## Value of the Data

• This dataset consists of sEMG signals of routine activities captured from upper limbs. These signals can be useful for differentiation of normal activity sEMG from strenuous activities sEMG like epileptic seizures, muscle fatigue etc.
• This database can be helpful in augmenting the already available databases which are specific to certain actions typically performed for gesture recognition. This can help researchers carrying out prosthetic development or seizure detection systems.
• The provided data can contribute towards development of assistive computer aided support systems for subjects with disabilities due to physical or mental disorder.
• The provided data can help improve the quality of life of people suffering from tonic-clonic epilepsy by providing a base line for normal activities.

## 1 Data Description

**Fig. 1:**
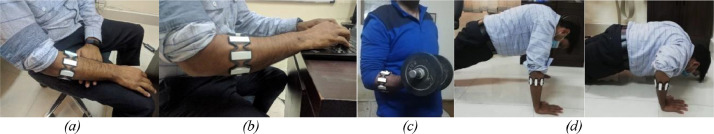
(a) Shows the subject during rest (b) typing activity (c) lifting of heavy object and (d) pushup activity.

Owing to the fact that sEMG can be captured in a non-invasive manner, these have certain repeatable patterns [2], and some classification methodologies [3, 4] make it possible to classify the signals in real-time, sEMG has applications in several different domains such as prosthetic development, human computer interface and gesture classification [1]. Therefore, the sEMG signals present in this dataset can play an important role to augment the already available datasets for better classification of such signals. sEMG database of routine activities is a dataset of 16 EMG signals of 70 s each saved as a comma separated values (.csv file). The signals have been captured using Myo Thalamic Armband from a single individual having no discernible disability. The subject was asked to perform 4 routine activities namely, resting, typing; push up exercise and lifting a heavy object. For the resting activity, the data was collected when the subject was at rest with the arms at the side supported below the elbow. Push-ups were performed on a level surface and were spaced out to accommodate the entire duration of the activity. Typing was performed with the keyboard placed at a level surface and the sentence “The quick brown fox jumped over the lazy dog” was typed over and over again. Lastly, a uniformly shaped heavy object weighing 20 kgs was lifted and held in air for the complete period of the activity. These activities are shown in Fig. 1.

**Fig. 2:**
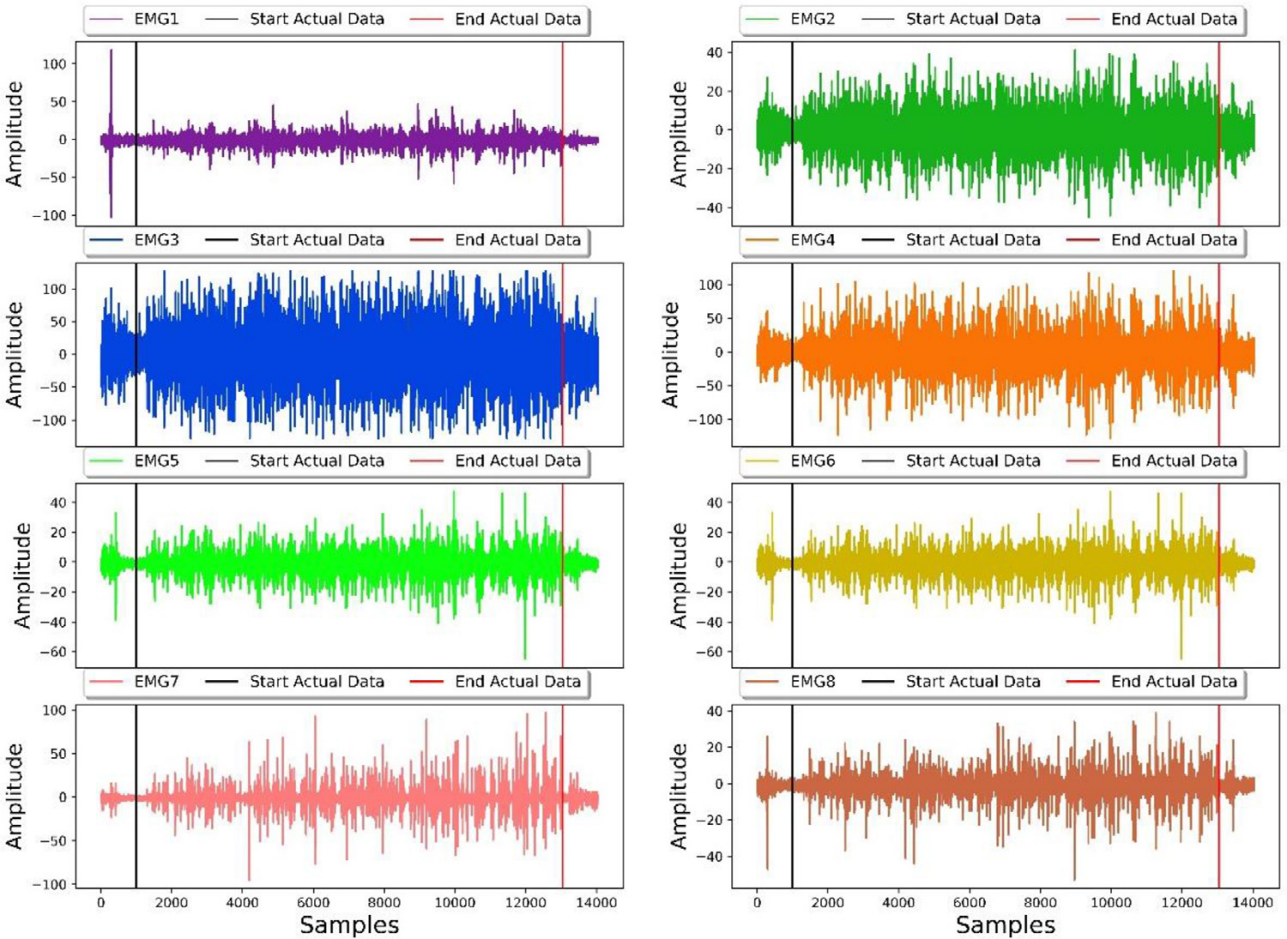
Sample 8 channel recording of sEMG for typing activity.

Each activity was repeated 4 times and sEMG signal has been saved as separate file. A rest period of 3 min was allowed between each repetition of an activity. During the resting period, the subject is asked to place their arm in a relaxed state with the palm facing downwards. Fig. 2 shows a sample sEMG signals against typing activity for 8 channels from this database where all the sEMG has been plotted against time. For each activity, the first 5 s and the last 5 s of data is invalid for that activity. These 5 s on both ends have been provided as a buffer. Both of these intervals can be visualized with help of vertical black and red lines drawn on original Fig. 2.

In addition to the EMG data, for each sample, the accelerometer data and gyroscope data has also been captured in separate .csv files. Using the gyroscope data, orientation has been computed in both Euler and non-Euler forms using set of Eqs. (1) and 2.

(1) $$\left[\begin{array}{c} x\\ y\\ z\\ w \end{array}\right]=\left[\begin{array}{c} v_{x}\sin\left(0.5\;\theta \right)\\ v_{y}\sin\left(0.5\;\theta \right)\\ v_{z}\sin\left(0.5\;\theta \right)\\ \cos\left(0.5\;\theta \right) \end{array}\right]$$

where $\left[\begin{array}{c} v_{x}\\ v_{y}\\ v_{z} \end{array}\right]$ is the unit vector representing the axis of rotation while θ is the angle of rotation.

(2) $$\begin{array}{ccc} roll & = & tan^{-1}\left(2*\left(w*x+y*z\right),\;-1*\left(x^{2}\;+y^{2}\right)\right)\\ pitch & = & sin^{-1}\left(\text{max}\left[-1,\;\min\left(1,\;2*\left(w*y-z*x\right)\right)\right]\right)\\ yaw & = & tan^{-1}\left(2*\left(w*z+x*y\right),\;-1*\left(y^{2}\;+z^{2}\right)\right) \end{array}$$

**Fig. 3:**
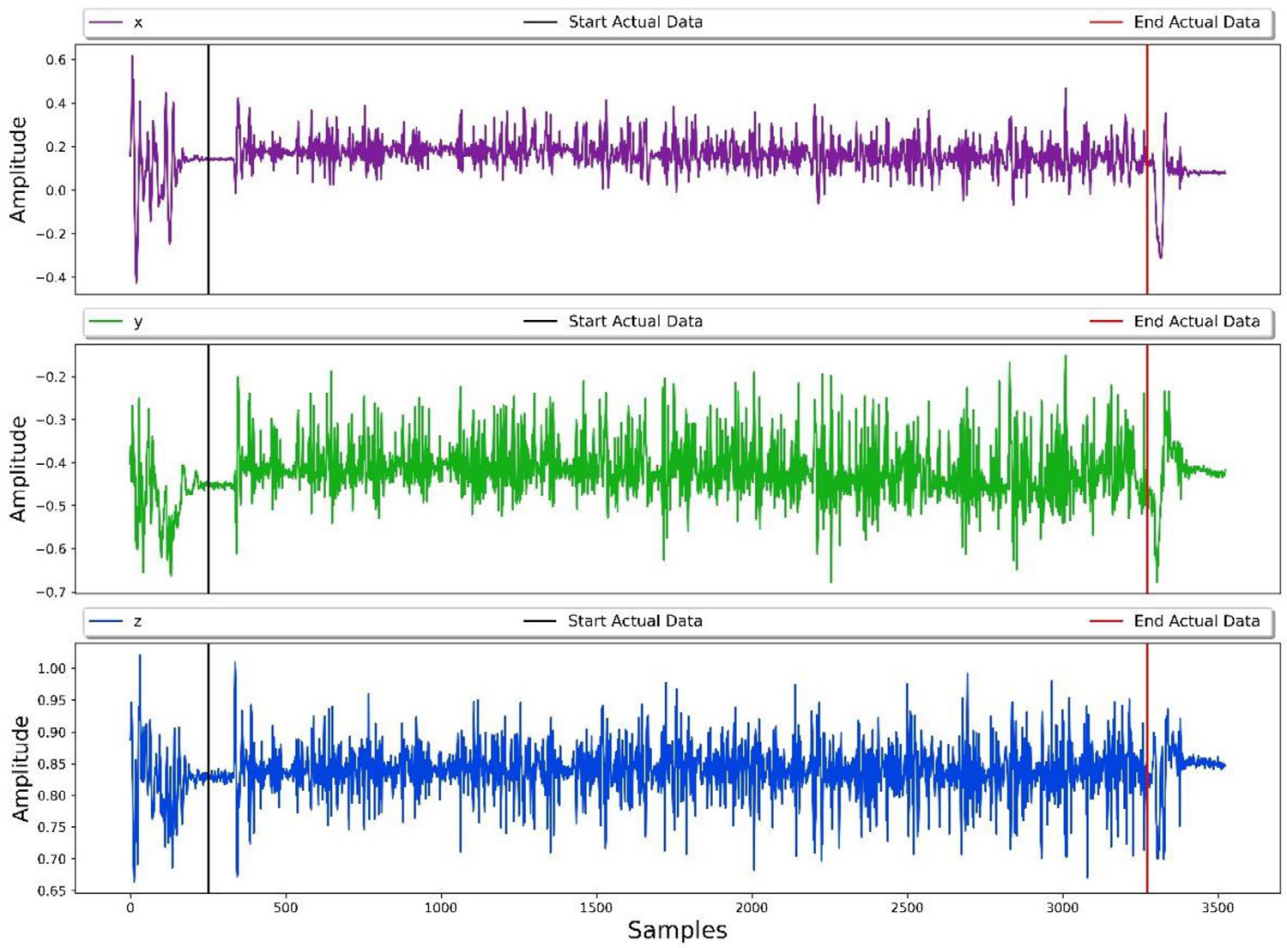
Sample recording of 3-axis Accelerometer signals for typing activity.

**Fig. 4:**
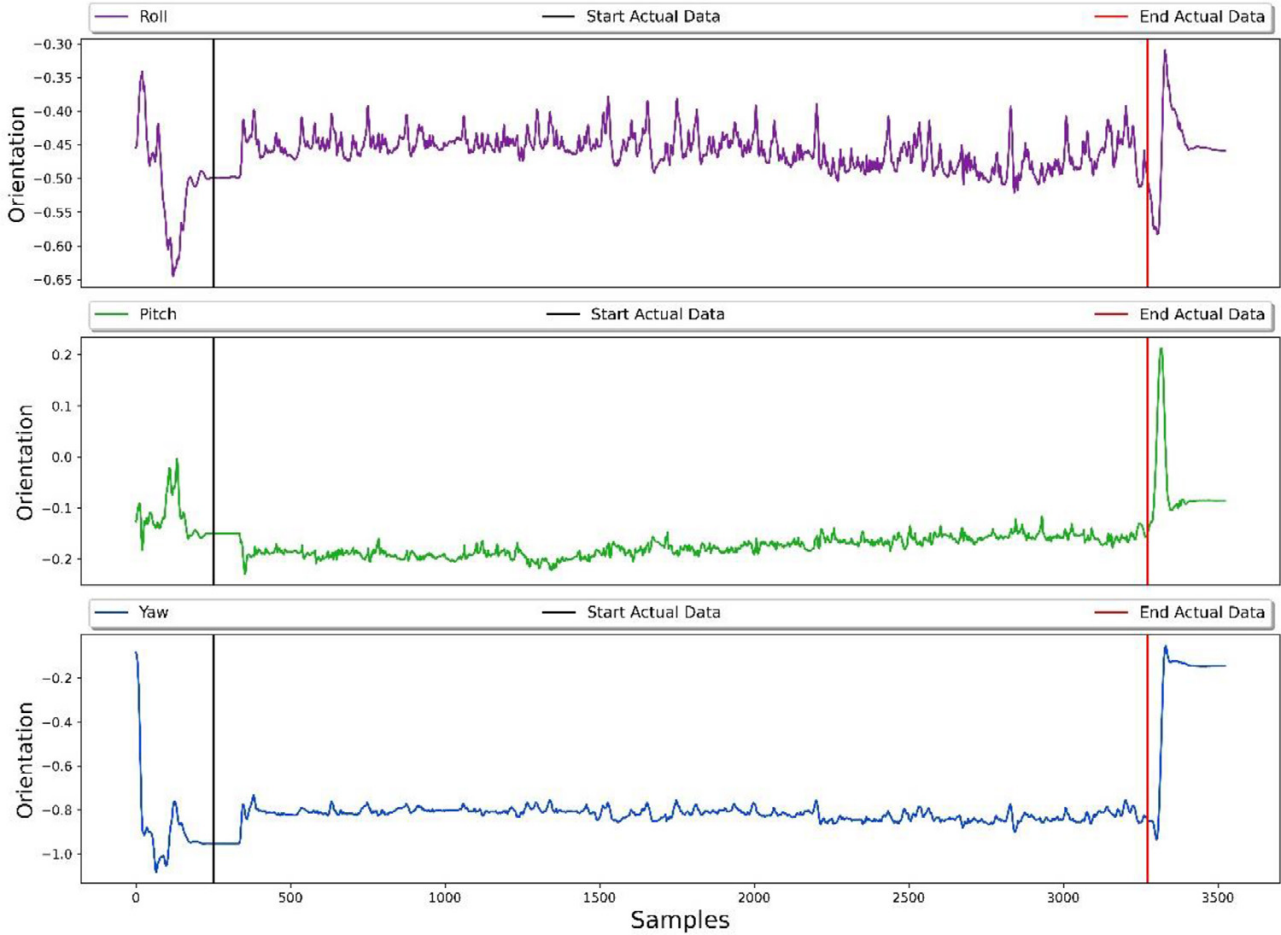
Sample recording of processed gyroscope signal in the form of roll, pitch and yaw for typing activity.

The processed values from gyroscope using Eqs. (1) and 2 are saved as a separate .csv file. A sample of both accelerometer and processed gyroscope values in the form of roll, pitch and yaw are shown in Fig. 3, Fig. 4 respectively.

Eventually, for each sample activity there are 5 associated files. The information from the accelerometer and the gyroscope can be coupled with the sEMG data [5] for better classification.

## 2 Experimental Design, Materials and Methods

**Fig. 5:**
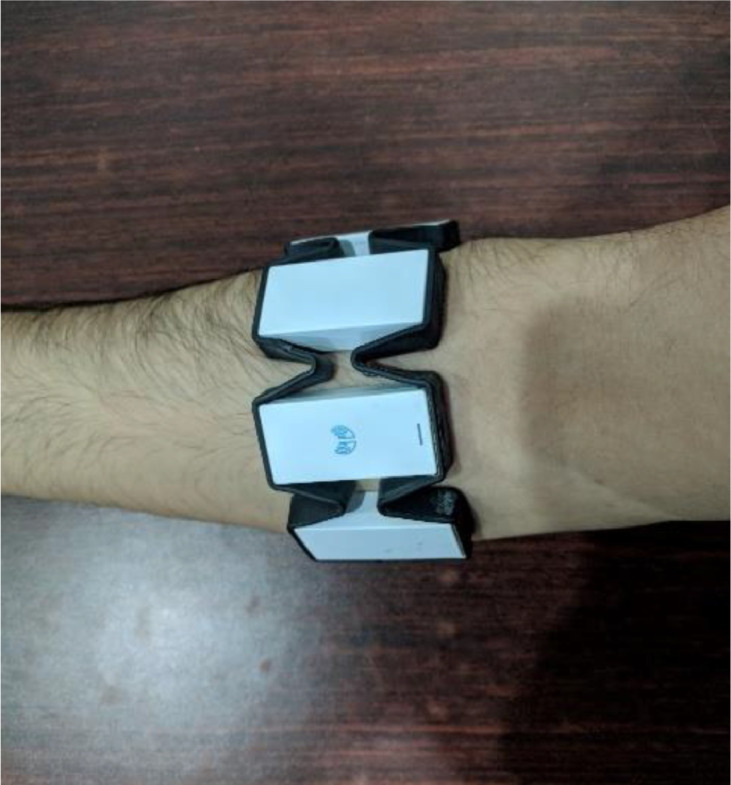
Placement of band on subject's upper limb below the elbow.

Myo Thalamic Armband [6] was used to capture the sEMG signals along with the associated accelerometer and gyroscope data. This particular band consists of 8 electrodes in a ring topology and captures the EMG signal in 200 Hz spectrum. The sampling frequency differs for the sEMG and the accelerometer and the gyroscope data, sEMG has been captured at 200 Hz sampling frequency while the latter have been captured at 50 Hz sampling frequency. Signed 8 bit values have been used to store the data captured by the band. The band transmits the data wirelessly to a Bluetooth dongle that is connected to a laptop. Once the band has synchronized with the dongle, the data is saved in the form of separate .csv files. The signals in this dataset have been acquired just below the elbow as recommended by [6] on the upper limb. Fig. 5 shows the placement of the band on subject's upper limb.

The data collection process with wearing the armband below the elbow as shown in Fig. 3. Before performing the actual activity, the subject is asked to relax for the first 5 s, which is categorized as the setup time. After the completion of activity, the data is recorded for an additional 5 s which acts as a buffer zone similar to setup time. As the band uses dry electrodes, there is no need to apply conductive gel on the skin for signal acquisition. However, the band needs to warm up when it is worn for the first, which may take up to a minute. A band-pass filter with cutoff frequencies 5–100 Hz is applied on the acquired sEMG signals, to minimize the effect of environmental noise and muscle movement, before storage and analysis. The processed sEMG values along with accelerometer and gyroscope signals are logged against each activity.

## Ethics Statement

All data was collected in a controlled environment with the consent of the subject where they agreed to volunteer for this data collection to support research. Subject filled a consent form before data collection. The authors have also received approval from department ethics committee for this research and data collection. The data provided online is fully anonymized and contains no information, which can reveal the identity of subject.

## Declaration of Competing Interest

All authors declare that they have no known competing financial interests or personal relationships which have, or could be perceived to have, influenced the work reported in this article.
